# Alpine ecology, plant biodiversity and photosynthetic performance of marker plants in a nitrogen gradient induced by *Alnus* bushes

**DOI:** 10.1186/s12898-020-00292-9

**Published:** 2020-04-20

**Authors:** Rexha Kaltrina, Bego Kristi, Zyruku Dea, Shuka Lulezim, Husi René, Schneller Jakob, Bachofen Reinhard

**Affiliations:** 1 Faculty of Natural Sciences, University of “Hasan Prishtina”, Prishtina, Kosovo; 2 Faculty of Natural Sciences, University of Tirana, Tirana, Albania; 3 Dept. of Evolutionary Biology and Environmental Studies, University of Zürich, Zürich, Switzerland; 4 Dept. of Systematic and Evolutionary Botany, University of Zürich, Zürich, Switzerland; 5 Dept. of Plant and Microbial Biology, University of Zürich, Zürich, Switzerland

**Keywords:** *Alnus*, *Calamagrostis*, Dwarf shrubs, *Frankia*, Plant frequency, N-indicator, Fast chlorophyll fluorescence, Nitrogen gradient

## Abstract

**Background:**

Alpine alder vegetation acts upon the nearby grass and dwarf shrub vegetation by the nitrogen supply from the symbiotic bacteria *Frankia alni* of *Alnus viridis.* This has been studied in two transects concerning plant distribution, plant diversity, nitrate concentration in soil and photosynthetic performance of specific marker plants.

**Results:**

Away from the alder stand, a band of some meters was dominated by *Calamagrostis varia* which then was followed by alpine dwarf shrub vegetation. Nitrate in the soil showed a concentration decrease away from the alder stand leading to values near the detection limit in the dwarf shrub zone. Within these three zones, plant species were distributed according to their N-index, given in the ecological literature. Three dominant species, *Calamagrostis varia*, *Rhododendron ferrugineum* and *Vaccinium myrtillus* were examined at sites of different N-availability in the horizontal transect for their photosynthetic performance, by measuring the prompt fluorescence, the OJIP named polyphasic rise of chlorophyll-*a* fluorescence. All three plant species showed signs of stress in the fluorescence rise kinetics at decreased nitrate availability. These are similar to other known stress effects such as faster reduction of the primary acceptor or an electron supply limitation on the donor site of photosystem II.

**Conclusion:**

Prompt chlorophyll-*a* fluorescence data of the examined leaves in a natural vegetation system showed the effects of a decrease in the essential nutrient nitrogen and in a manner parallel to changes in plant diversity. The selected marker plants behaved differently towards decreasing nitrogen concentrations in soil.

## Background

Nitrogen is one of the main nutrients for plant growth and is considered to be the limiting factor for net primary production in terrestrial ecosystems [34, 35]. For high productivity in agriculture, nitrogen is supplied as fertilizer in the form of nitrate or ammonium. In natural pristine environments nitrogen fixing bacteria, free living or as root symbionts, provide bound nitrogen in nitrogen-poor soil. In the alpine shrub and grass zones the concentration of available nitrogen is often low; although various nitrogen compounds may be imported through the atmosphere by wind or rain. *Alnus viridis* is an important component of the tall shrub vegetation in the subalpine and alpine areas. Due to root symbiosis with the nitrogen-fixing Actinobacterium *Frankia alni*, the soil at these sites is enriched in plant-available nitrogen and a remarkable accumulation of biomass may be seen nearby. This suggests the presence of a nitrogen flow away from the alder zone into the neighboring grass and shrub zones. Thus, a clear change in plant composition and diversity away from the border of the *Alnus* tall forbs is often observed even with the naked eye.

**Fig. 1:**
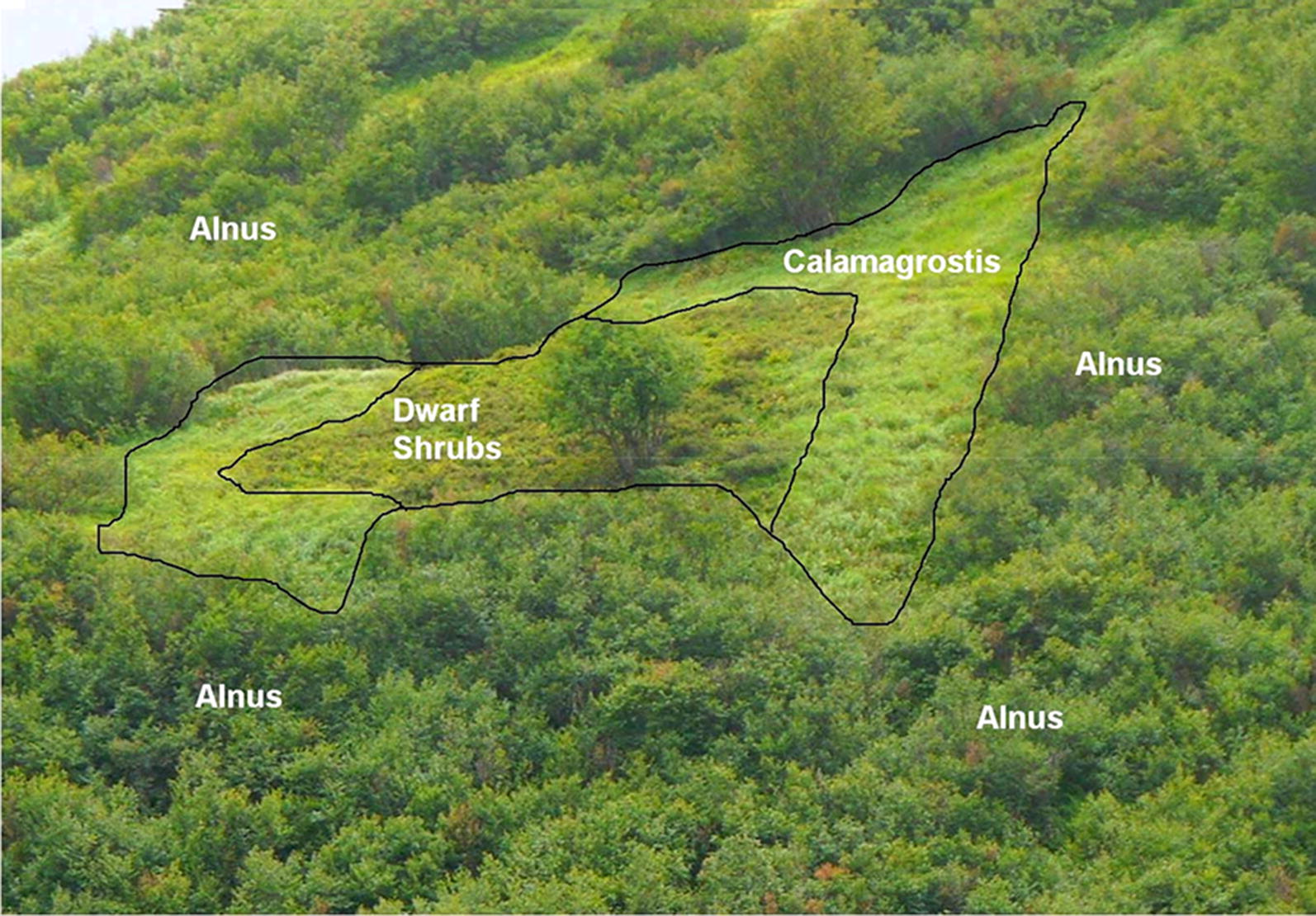
Photo of the experimental site with the *Alnus viridis* zone, the *Calamagrostis varia* band and the dwarf shrub zone. (Photo: J. Schneller)

**Fig. 2:**
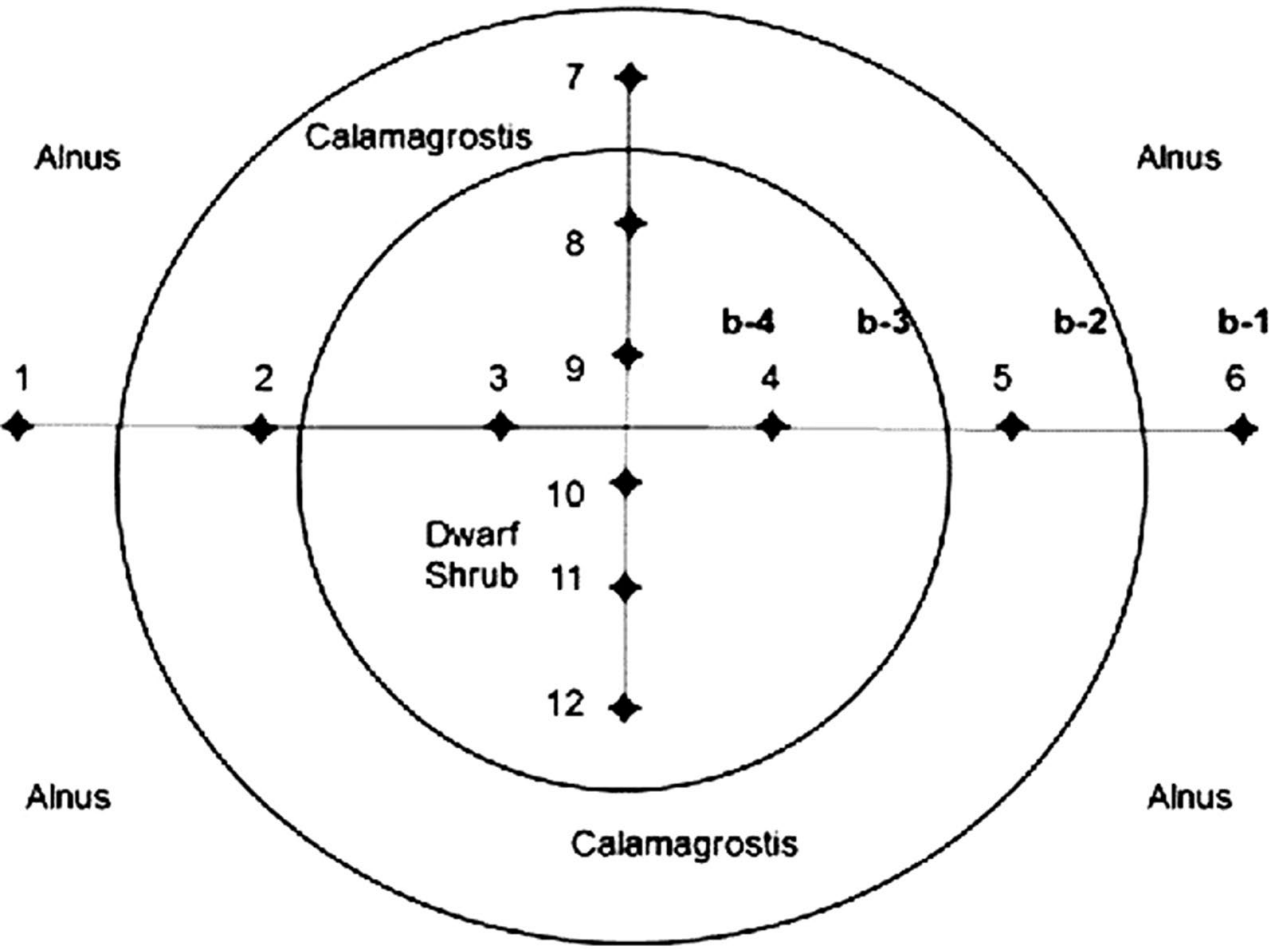
Schematic drawing of the experimental site with the three main vegetation zones characterized by the dominant marker plants species *Alnus, Calamagrostis* and dwarf shrubs. The squares of 1 m^2^ used for floristic investigation are arranged as horizontal (1–6) and vertical (7–12) transects. The locations of the sampling of soil for nitrate determination and of the plant leaves for fluorescence measurements are labeled as b-1 to b-4; b-5 is some 100 m away from *Alnus* trees in a dwarf shrub area

In the Piora valley (Central Switzerland, Canton Ticino) such sites are found on the north- exposed slope of the Mottone, south of the Murinasca River. In an open area between *Alnus* bushes and trees a defined stripe of grasses (mainly *Calamagrostis*) is observed growing adjacent to the *Alnus* border (see Figs. 1, 2). This grass vegetation is abruptly followed by dwarf shrubs, suggesting that sudden changes in nutrient supply might be the cause. It is known that nitrogen-species are washed out from the soil at *Alnus*-dominated sites [6] and from nitrogen-enriched forest [26]. Plant biodiversity changes along the nitrogen gradient. To quantify these observations, the plant composition and diversity were studied in a horizontal and a vertical transect perpendicular to the *Alnus* bushes. The effect on plant metabolism was studied by measuring the photosynthetic performance of selected dominating plant species at different sites in the N-gradient.

In recent decades, chlorophyll-*a* fluorescence techniques have been used to measure the fitness of or the stress upon plants caused by pollutants (e.g. ozone, [3]), but also by environmental climatic factors such as heat and drought, or the availability of nutrients. These techniques are non-invasive and rapid, and ideal for field measurements, as reviewed recently by Kalaji et al. [13]. After dark adaptation to obtain a fully oxidized acceptor site of photosystem II, a strong light flash increases chlorophyll-*a* fluorescence from a minimal value at time 10 μs (O = F_o_ = initial fluorescence) to a maximum at 0.3 to 1 s (P = F_m_ = maximal fluorescence). When plotted in a logarithmic scale, this transient shows polyphasic kinetics, with steps at 2 ms (J-step) and 30 ms (I-step) (see kinetics in Fig. 5). This sequence has been named the OJIP-test. With a detailed analysis of it, specific information can be gained to estimate the vitality of plants [30, 31]. The F_v_/F_m_ value is frequently used in the literature, designating the maximum quantum yield of the photosystem II, F_v_ being the variable fluorescence (F_m_-F_o_). However, F_v_/F_m_ covers only primary photochemistry as originally discovered by Butler and Kitajiama [4]. This parameter therefore has been found to be relatively insensitive towards many metabolic changes in the plant induced by environmental effects. Several other parameters can be derived from the OJIP kinetics which seem to be more sensitive and more specific than the F_v_/F_m_ value (e.g. [2, 12, 18, 23, 24, 36, 37]). The level of the minimum fluorescence F_o_, the initial velocity of the fluorescence rise, or the J-point, the fluorescence level reached after 2 ms have been suggested as valuable markers. Such factors are pooled in the photosynthetic performance index PI. Thus the PI covers more information by combining primary photochemistry with dark reactions of the photosynthetic electron transport and the CO_2_-fixation downstream the charge separation in photosystem II. For a detailed discussion on the theory behind the OJIP-test see Strasser et al. [31] and Stirbet et al. [29].

In various crop plants, the OJIP-fluorescence induction kinetics is influenced not only by physical environmental stress, but also by the availability of various nutrients, among these nitrogen [19, 25, 33, 37, 38]. In controlled nitrogen fertilization experiments with a single plant species of same age and genetics, differences in photosynthetic performance related to nutrient concentration have been demonstrated. In this study we present the effect of different nitrogen availability in soil—produced by a natural gradient caused by nitrogen leaching from *Alnus* vegetation—on the photosynthetic performance of typical common plants in a natural alpine ecosystem. These results correlate well with ecological changes in plant species distribution and biodiversity at these sites and with the empirically determined N-index values in the literature.

## Results

### Characterization of vegetation and environment

The vegetation assessment demonstrates the change of species within their habitat along the two transects (see Fig. 2). Table 1 shows the floristic composition of the plant species in each of the 6 plots of the horizontal and the vertical transect with the semi-quantitative estimation of the plant frequency based on Braun-Blanquet [1]. The scaling runs from 1 = very few to 6 = dominant. Plant distribution was characterized by three clearly distinct zones with increasing distance from the *Alnus* vegetation, first a *Calamagrostis* belt (1 to 3, 7, 8) and then the dwarf shrub zone in the center (4, 5 and 9 to 12). These zones were easily distinguished by eye, using vegetation structure and the intensity of leaf color, even within the same species in different parts of the transect. *Rhododendron ferrugineum* was hardly found near the *Alnus* bushes, while *Vaccinium myrtillus* or *Veratrum album* were spread over the whole transect. Besides *Rhododendron*, several genera such as *Oxalis, Homogyne* or *Gentiana* seemed to avoid sites close to the *Alnus* zone.

**Table 1:** List of plants and their frequencies observed in the horizontal (sample 1–6) and vertical transect (sample 7–12)

Number of plots	L-val.	Horizontal plots						Vertical plots					
		1	2	3	4	5	6	7	8	9	10	11	12
Species													
*Achillea macrophylla*	4		+										
*Alnus viridis*	4	4					4						
*Athyrium distentifolium*	3		2										
*Avenella flexuosa*	2				+				+	+	+	2	+
*Calamagrostis varia*	2	1	1			2		4					
*Dryopteris dilatata*	3	+		+		+		+			+		
*Festuca rubra*	3						+			+			
*Gentiana purpurea*	2				1	+	+			+	+		
*Homogyne alpina*	2		+		1	2	+		+			+	
*Juniperus communis*	2					+							
*Ligusticum mutellina*	3		+										
*Lilium martagon*	3		+										
*Luzula silvatica*	2										+		
*Oxalis acetosella*	2	+	1	+	1			1	+	+	+	+	+
*Phleum alpinum*	2						+						
*Rhododendron ferrugineum*	2	+		1	2	2	+		+	2	3		5
*Solidago virgaurea*	3						+						
*Sorbus aucuparia*	2					+		+					
*Vaccinium myrtillus*	2	1		+	2	3	1		+	1	+	3	+
*Vaccinium uliginosum*	2				3	2				3	3	3	
*Veratrum album*	4	1	4	1		1	3	3	1		+		+

The frequency of the species found is given following the cover abundance scale of Braun-Blanquet [1], ranging from +: 2%, 1: 2–5%, 2: 5–25%, 3: 25–50%, 4: 50–75%, 5: 57–100%. L-val. = Landolt N indicator value Landolt et al. [17]

**Fig. 3:**
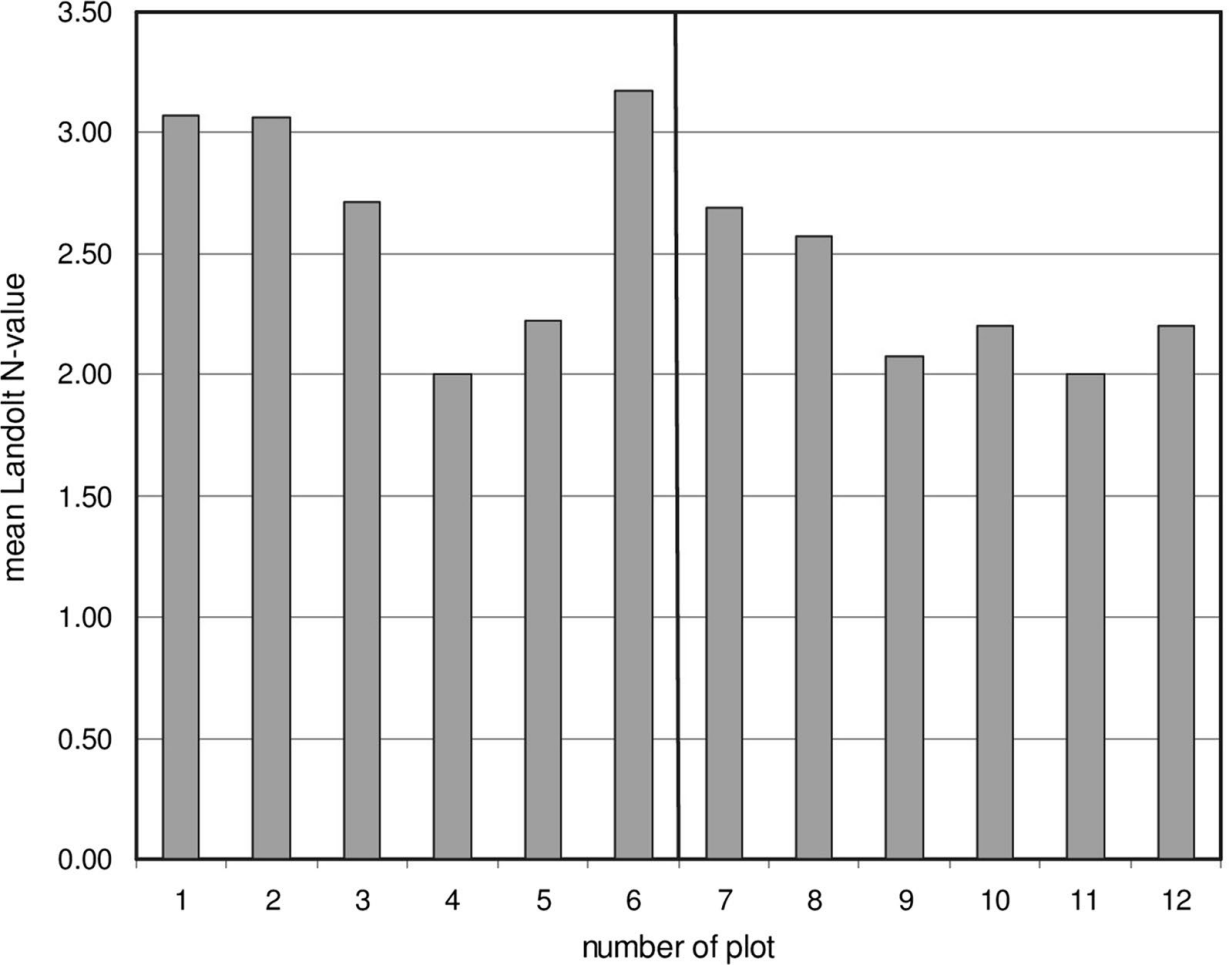
Mean of indicator values calculated from plant frequency [1] and Landolt indicator value [17] for the 12 sampling plots in the horizontal (1–6) and the vertical (7–12) transects (see Fig. 2)

N-indicator values [17] characterize the preference of a plant towards available nitrogen in soil. Combined with the frequency of species in each sample plot (given in column 1 of Additional file 1: Table S1), a N-indicator value for plant-available nitrogen at each site was calculated (see Additional file 1: Table S1). The mean of the indicator values of all species collected suggested the presence of a nitrogen gradient, with highest values in the surrounding *Alnus* zone (Fig. 3, plots 1, 2, 6, 7), then decreasing towards the open part, with the lowest values within the dwarf shrub area (plots 3, 4, 9–12). On the same basis the plant composition indicated that the pH value drops from the *Alnus* zone towards the dwarf shrub zone (not shown). For topological reasons the effect of *Alnus* was highest in plot 6 which was situated within the *Alnus* stands.

### Available nitrogen in soil

**Fig. 4:**
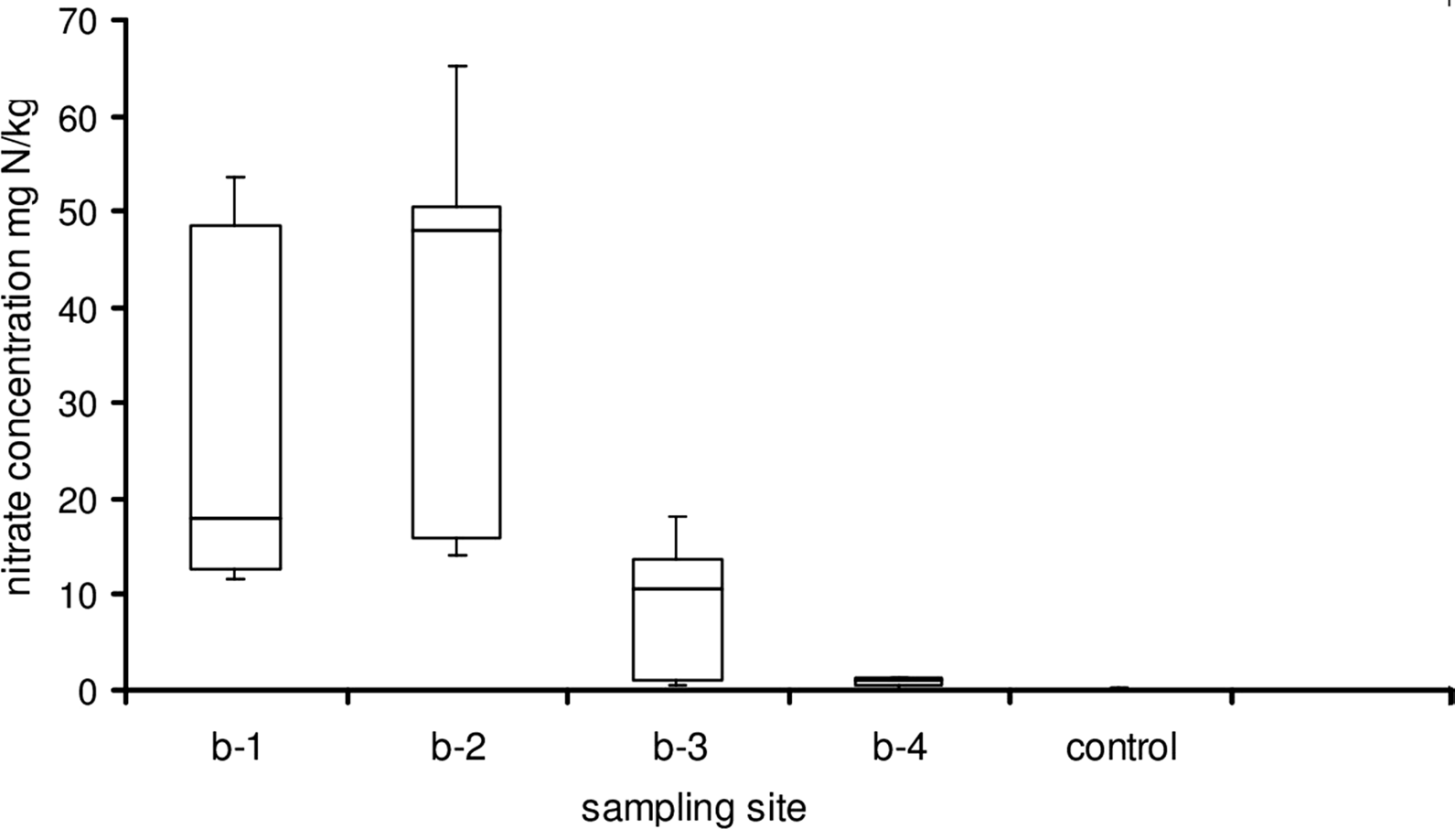
Nitrate concentration in soil (mg nitrate–N/kg dry soil, for the sampling sites see Fig. 2). Box plot with median value, 25th and 75th percentiles and minimum and maximum values (n = 5, control = sampling site b-5). For statistical treatment see text and Additional file 2: Table S2

To quantify differences in N-concentration in the soil, nitrate as the plant-available nitrogen was determined in soil samples at sites b-1 to b-5. In the well-aerated alpine soil, only nitrate was detected as plant-available nitrogen. These data confirm that nitrate was washed-out from the *Alnus* site into the dwarf shrub zone (Fig. 4). As the soil was rich in particulate organic fragments and inhomogeneous, the nitrate content varied broadly within samples from the same plot. Differences between the *Alnus* site (b-1) and the *Calamagrostis* zone (b-2 and b-3) were statistically not significant (n = 5, significance level 5%). The values of sites b-1 and b-2 were statistically different from b-4 and b-5 (= control); b-3, as well, was statistically different from b-5; see Additional file 2: Table S2.

### Photosynthetic activity measured by the prompt fluorescence

**Fig. 5:**
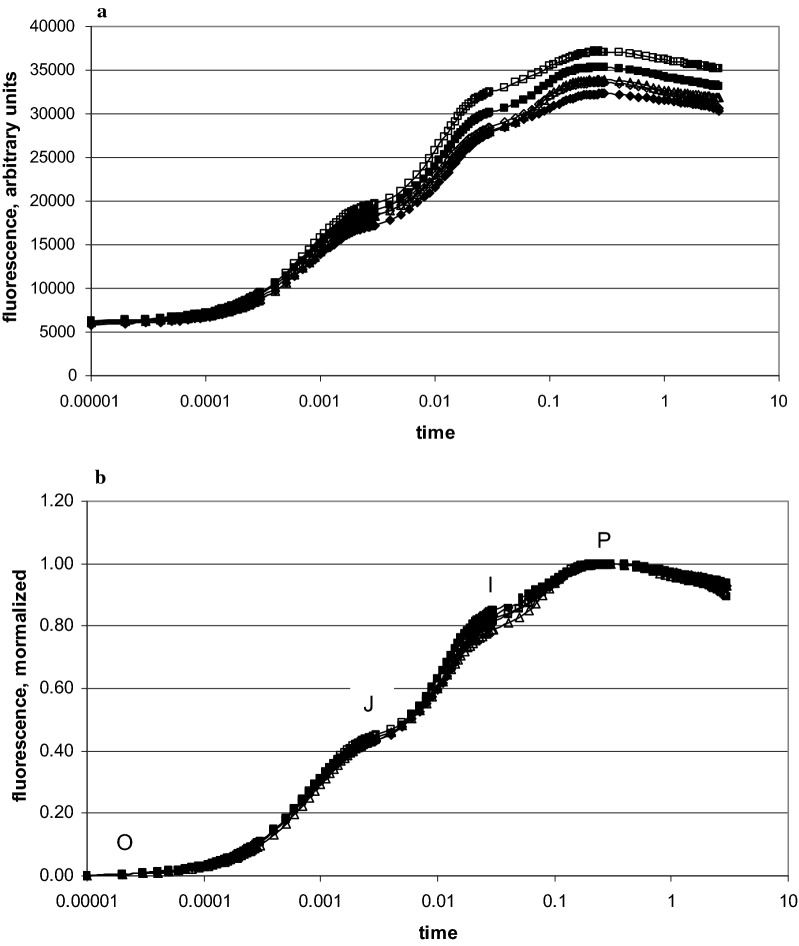
Fluorescence induction scans from 10 μs to 3 s of 5 different *Alnus* leaves from site b-1, **a** raw data, **b** the same data after double normalization between F_o_ = 0 and F_max_ at 0.3 s = 1. All data points are means of 15 measurements (data of 2. August 2017)

**Fig. 6:**
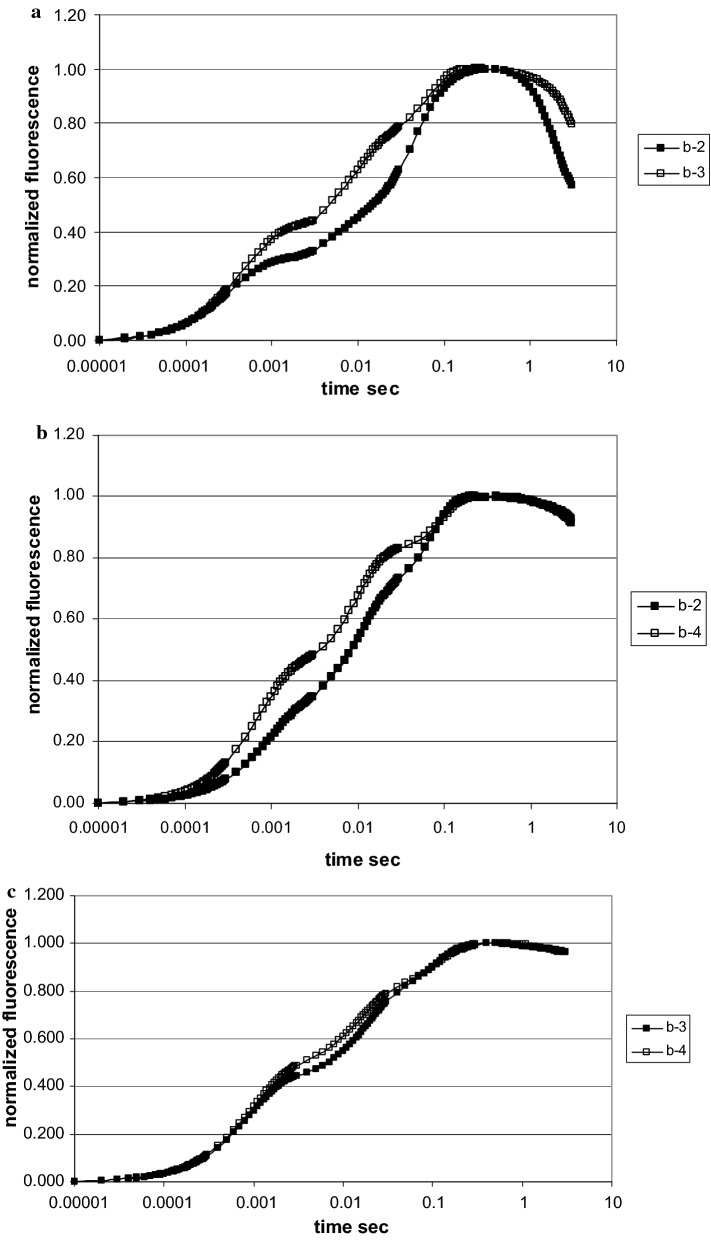
Fluorescence induction scans from 10 μs to 3 s after double normalization between F_o_ = 0 and F_max_ at 0.3 s = 1, for *Calamagrostis* (sites b-2 and b-3), *Rhododendron* (sites b-2 and b-4) (data of 2. August 2017) and *Vaccinium* (sites b-3 and b-4) (data of 13. September 2017). All data points are means of 15 measurements

The raw fluorescence kinetics of all plant species at all sites exhibited the typical polyphasic OJIP fluorescence rise. Raw data (Fig. 5a) often showed large differences in the fluorescence intensity. This was due to the variability between the various plants of the same species at different sites as well as within their leaves. While the mean value of the F_o_ stayed at about the same level (see also Tables 2, 3), the highest F_m_ in single runs was up to twice that of the lowest one in plants in the heterogeneous shrub zone, due to leaf age and local soil conditions. For further analysis, F_o_ and F_m_ data were double normalized between the time 10 μs for F_o_ = 0 and 0.3 s for F_m_ = 1 (Fig. 5b). This allowed searching for individual differences within the induction phase. By averaging the data of 15 measurements for the same plant species and location, specific environmental effects in photosynthetic performance at different sites became in evidence. The data of the three selected plants, *Calamagrostis, Rhododendron* and *Vaccinium,* demonstrated kinetic differences relative to the nitrate concentration in the soil. In summary all tested plant species displayed a more rapid fluorescence rise at lower nitrate, indicating a faster closure of the primary acceptor of PSII and a reduced electron flow beyond. In *Vaccinium* the differences between lower and higher nitrate availability were small and only visible in the JI part of the fluorescence rise. In *Rhododendron* the two traces had already separated by ~ 0.2 ms, while in *Calamagrostis* they splitted only after 3 ms (Fig. 6).

**Table 2:**
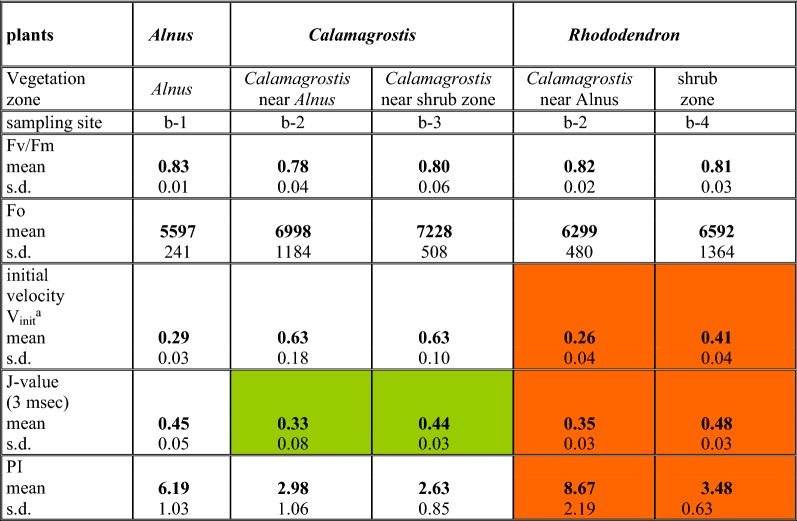
Selected parameters from OJIP prompt fluorescence rise (data of 2. August 2017) for the species *Alnus, Calamagrostis* and *Rhododendron*

For *Calamagrostis* and *Rhododendron* the data at the two sites in the nitrate gradient (b-2 with b-3 and b-2 with b-4, respectively) are compared. All numbers are means of 15 measurements. Colored squares identify the plants for which the results are statistically different (p < 0.01) at the two sites

^a^the initial velocity has been calculated as V_init_ = F_V150 μsec_/0.15

**Table 3:**
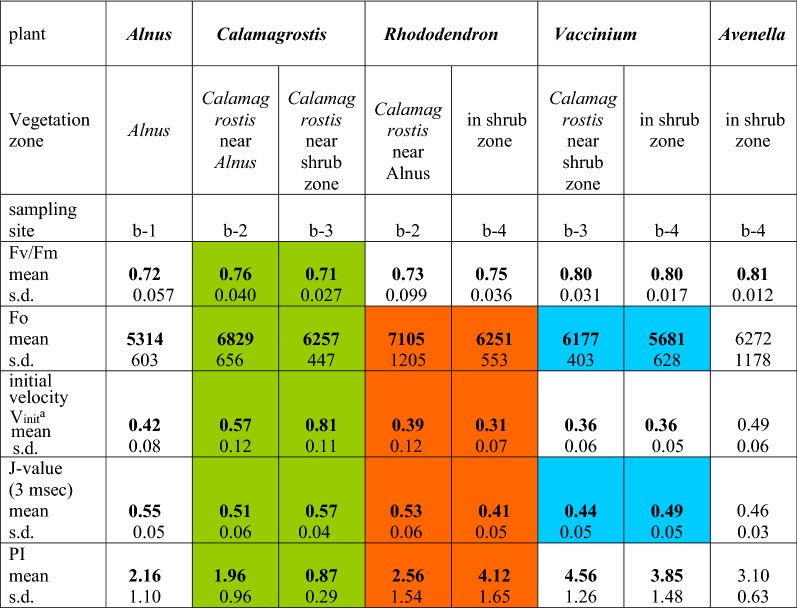
Selected parameters from OJIP prompt fluorescence rise (data of 13. September 2017) for the species *Alnus, Calamagrostis, Rhododendron, Vaccinium* and *Avenella*

For *Calamagrostis, Rhododendron* and *Vaccinium* the data at the two sites in the nitrate gradient (b-2 with b-3, b-2 with b-4 and b-3 with b-4, respectively) are compared. All numbers are means of 15 measurements. Colored squares identify the plants for which the results are statistically different (p < 0.01) at the two sites

^a^the initial velocity has been calculated as V_init_ = F_V150 μsec_/0.15

Several parameters can be extracted from prompt fluorescence; they give quantitative informations and can be treated with statistical tools.

Tables 2, 3 list some parameters which others have described as being affected by environmental impacts. They are compared pairwise for the same plant at different N-contents in the soil.

The most frequently used parameter so far in the literature to quantify the photosynthetic performance is the F_v_/F_m_ value, the maximum yield of primary photochemistry. In healthy plants it is around 0.8, but many environmental effects may lower it substantially. The mean values of 15 measurements in such a diverse habitat type spanned the range for all plants and locations from 0.71 to 0.83; and these values were significantly lower mid-September as compared to beginning of August. In early August the F_v_/F_m_ values of *Alnus*, *Calamagrostis* and *Rhododendron* stayed high around 0.8; 6 weeks later all values had dropped. As in summer the highest values were observed in fall with *Vaccinium* and *Avenella* in or near the shrub zone. Regarding a nitrogen effect, only *Calamagrostis*, in fall, showed a higher F_v_/F_m_ value near the *Alnus* zone (b-2) as compared to the shrub zone (b-3). The difference is small, but statistically significant with p < 0.01. In contrast *Rhododendron* had slightly lower but not significant values near the *Alnus* zone (b-2) as compared to in the shrub zone (b-4). For *Vaccinium* no statistical difference was observed between the two sites and the value for *Avenella* was similar to *Vaccinium*.

The initial fluorescence, F_o_, has also been suggested to respond to environmental signals: it rises at stress situations, e.g. after long drought periods [9]. The F_o_ variation observed along the transect ranged mid September from 5314 counts for *Alnus* to 7228 in early August for *Calamagrostis* near the shrub zone. In early August there was no difference between the two sites, neither for *Calamagrostis* nor for *Rhododendron*. In contrast, in mid September the level of F_o_ in both species was statistically different for the two sites (p < 0.01), interestingly both species with a higher F_o_ near the *Alnus* zone with more nitrogen. For *Rhododendron* the value near *Alnus* (b-2) was statistically different from the one sampled in the shrub zone (b-4); also the F_o_ values for *Vaccinium* are different for the two sites (b-3 and b-4) (p < 0.02).

As shown in Fig. 6, for both *Calamagrostis* and *Rhododendron,* the initial fluorescence rise was different between plants near the *Alnus* site as compared to those at some distant away. The initial slope dF_v_/dt is a measure of the initial rate of the closure of the RC of PS II; it increases when the electron transport beyond the primary acceptor of PS II is impeded. In summer no difference was seen for *Calamagrostis*, while *Rhododendron* reacted to limiting nitrogen by a more rapid initial increase. In September, with all species, the initial rise was faster compared to beginning of August; while in summer *Calamagrostis* had identical values for V_init_, it was in fall about 30% higher at the site near the shrub zone (b-3) compared to near *Alnus* (b-2). In *Rhododendron* V_init_ differed in summer with lower values at the site near *Alnus* (b-2). In contrast the situation was reversed mid September when the shrub zone (b-4) had a lower V_init_.

The J value, the normalized relative fluorescence level reached after 3 ms, is as well a measure of how fast the primary acceptor of PSII becomes reduced. *Calamagrostis* and *Rhododendron* presented both in summer and fall a better photosynthetic performance close to the *Alnus* zone with its higher nitrogen content in the soil. In contrast, in September the J-value of *Rhododendron* close to *Alnus* was clearly higher. The J-values of *Vaccinium* gave a better performance in *Calamagrostis* (b-3) compared to the site in the shrub zone (b-4).

The photosynthetic index PI is a parameter frequently used for measuring environmental stress effects, especially for monitoring the vitality of crop plants. Similar to the other parameters described above, the PI of *Calamagrostis* had dropped in fall. In summer the difference between higher and lower nitrate content (b-2 compared to b-3) was small but significant; the difference was double that of September (p < 0.001). In summer *Rhododendron* had quite higher values near *Alnus* (b-2) with a p < 0.001 compared to the site in the shrubs (b-4). For *Vaccinium* the two sites were statistically identical.

**Fig. 7:**
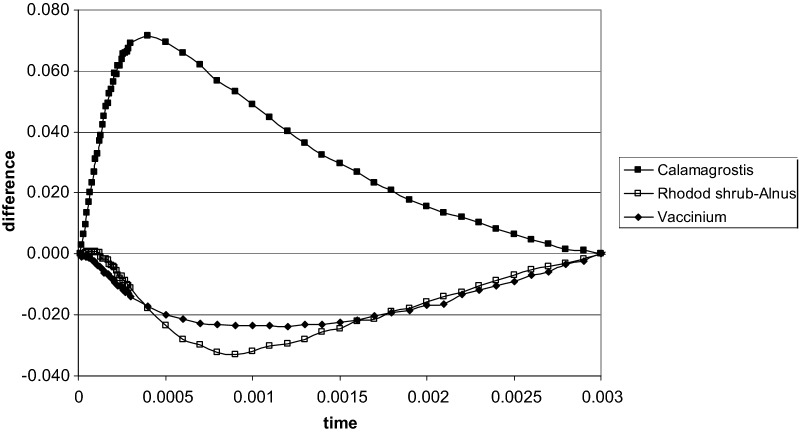
Differences in the fluorescence kinetics between sites of low and high nitrogen for *Calamagrostis* (sites b-3 versus b-2), *Rhododendron* (sites b-4 versus b-2) and *Vaccinium* (sites b-4 versus b-3) after double normalization between time 0 and 3 ms with linear time scale (K-band) (data of 13. September 2017)

Various stress situations cause specific differences in the early fluorescence rise at around 300 μs, the K band. In our experiments, as shown in Fig. 7, when the differences (within the first 3 ms) between the three species, *Calamagrostis*, *Rhododendron* and *Vaccinium*, close to and away from *Alnus*, are compared, clear differences are apparent: In *Calamagrostis* the signal (higher N–lower N) is positive, while in *Rhododendron* and *Vaccinium* it is negative with a delayed minimum at 1 ms.

**Fig. 8:**
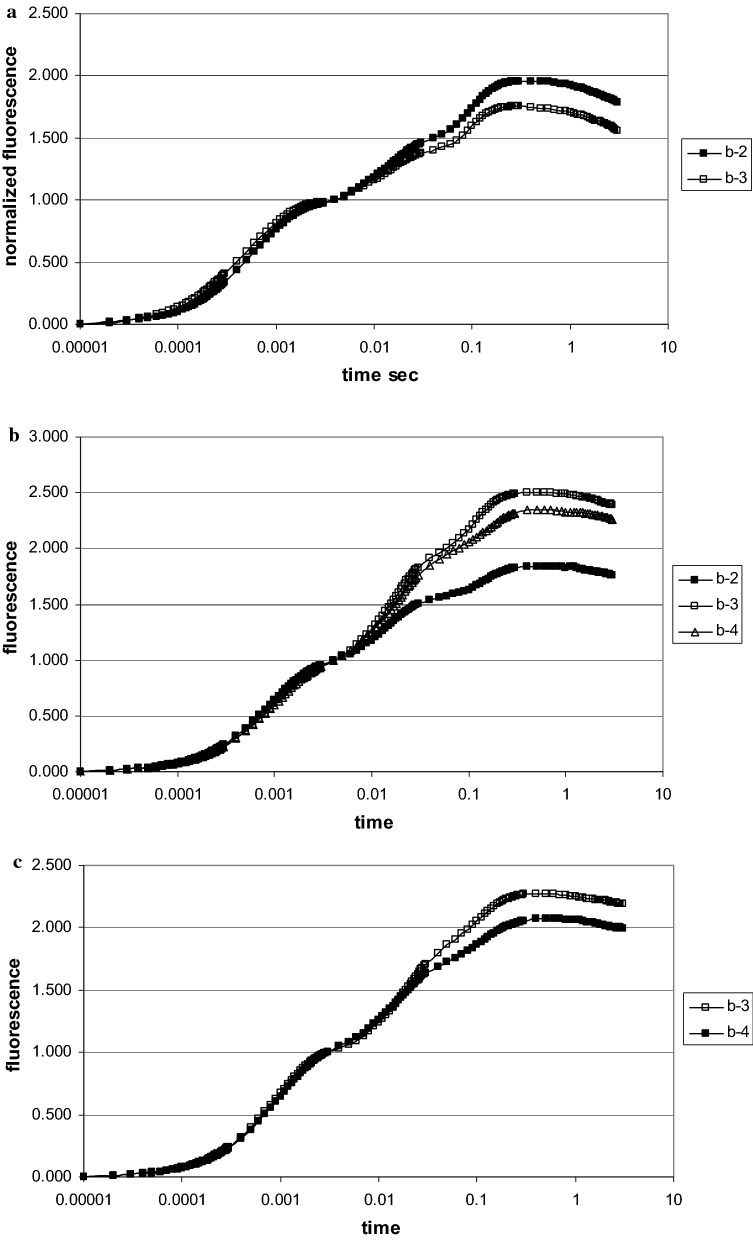
Fluorescence induction scans from 10 μs to 3 s after double normalization of the O-J phase between F_o_ = 0 and F_J_ at 3 ms = 1. *Calamagrostis* (sites b-2 and b-3), *Rhododendron* (sites b-2 and b-4) (data of 2. August 2017) and *Vaccinium* (sites b-3 and b-4) (data of 13.September 2017). All data points are means of 15 measurements (for comparison see also Fig. 6)

The final section of induction kinetic, the I-P part, gives some information on the electron transport from PS II to PS I and beyond. For all three tested species, clear differences were observed (Fig. 8). After normalizing between 0 and 3 ms (O-J phase), no differences between the sites of varying N-availability were apparent within this time period; the traces are superimposed. However, the fluorescence kinetics separated between about 10 and 100 ms. All three plants had a higher electron transport activity at the site of higher nitrogen supply (b-2) as compared to the site near the shrub zone (b-3, b-4).

## Discussion

*Alnus viridis* is widely present in the Swiss Alps at altitudes between 1500 and 2500 m a.s.l. and develops in many places when the alpine grass vegetation is not grazed by cattle or sheep. With its symbiotic partner *Frankia alni*, it affects the nitrogen supply in soil within and near the *Alnus* covered area, leading to a gradient of plant-available nitrogen away from *Alnus* stands. This has been confirmed by three observations: (a) analyses of nitrate in the soil (Fig. 4), (b) characterizing the plant diversity using the N-index from the literature (Fig. 3), and (c) interpreting the responses of the plant photosynthetic performance with fast fluorescence kinetics (Figs. 6, 7, 8).

The dispersal of different species along the two transects (2. August 2017) indicate the allocation of differently adapted species to the nitrogen content of the soil (Table 1). Plant indicator values have been empirically worked out for each species by comparing growth and ability for competition in relation to available nitrogen in soil under natural conditions [7, 16, 17, 32]. When combined with the plant frequency in each plot, these values correlate well with the actual nitrate concentration (Fig. 3). High values were found for plot 1, 6 and 7, all situated within the area of *Alnus* plants, while the values of the plots in the shrub zone are clearly lower.

The plant community composition has a strong influence on the retention as well as on the loss of nutrients on a local as well as in a watershed scale. Nitrate and dissolved organic nitrogen concentrations in small rivers are strongly related to the vegetation cover in its watershed. In a region in Oregon (USA) dominated by red alder, a massive leaching of nitrogen was observed [6]. Similarly, in our experiment *Alnus viridis* acts as a strong control on ecosystem function and plant diversity.

Effects of nitrogen supply on photosystem II photochemistry have been studied for various important crop plants [12, 13, 18, 19, 25, 33, 37] Nitrogen deficiency, as a physical environmental stress factor, acted on the fluorescence induction rise during the first second. These lab experiments were run with plants under controlled growth conditions and with a defined addition of the nutrient. Observed effects can be compared to a control situation and correlated to the stress imposed. In field experiments real controls are missing, in our experiments we compared plants of the same species pairwise at different sites in a gradient of nitrate in the soil.

The most frequently used parameter to measure stress effects or the photosynthetic performance of plants is the maximum quantum yield of PS II, the ratio of the variable fluorescence to the maximum one (F_v_/F_m_). However, compared with other indicators of photosynthetic performance, it has been found to not be very sensitive; no seasonal variation or response to drought was observed with beech saplings [22] or *Triticum* reacting only after long periods of dehydration [36].

Under nitrogen limitation the F_v_/F_m_ dropped in maize from 0.76 in the control to 0.68, while in tomato the effect was even smaller [11]. Li et al. [18] reported only minimal differences for maize at 2 different levels of nitrogen in soil. Similarly, while water logging for 20 days reduced leaf nitrogen in *Medicago sativa* to less than half, at the same time the F_v_/F_m_ value dropped by about 15% [27].

In our experiments the relative deviation to the mean values of the F_v_/F_m_ with 15 measurements for each plant and each site was between 1.2 and 13.5%, indicating some diversity between single plants within the same species and site. Tables 2, 3 present all data as mean values of 15 measurements. For all selected parameters white squares with the same species at the two sites of different soil nitrogen are statistically similar while colored fields are pairwise statistically different (t-test p < 0.01). Overall the F_v_/F_m_ values do not differ much in relation to the nitrate concentration, but they are clearly lower mid-September compared to early August. In early August the values do not differ pairwise. However, 6 weeks later, the two values of *Calamagrostis* (b-2 and b-3) differ statistically (p < 0.01). Similarly, in early August the two values of *Rhododendron* are identical, while in fall, the site near *Alnus* is statistically different from the site in the shrub zone. *Vaccinium* shows the highest F_v_/F_m_, but they are statistically identical. These data confirm that F_v_/F_m_ is only a rough measure of photosynthetic performance or vitality in a mixed plant population in an ecosystem of heterogenic soil and nutrient supply conditions.

The minimal fluorescence, F_o_, is said to be sensitive towards environmental extremes [5, 9, 13]. Would F_o_ also react on differences in nitrogen availability? In early August the differences in the level of F_o_ are small and pair wise statistically not significant, while in mid-September, the F_o_ of *Calamagrostis* at the two sites is different with a p < 0.01. For *Rhododendron* and *Vaccinium* statistical differences are found between the site near *Alnus* and the one more distant. However, in all cases the site of higher nitrate resulted in a higher level of F_o_, which is in contrast to the expectation that stress will increase F_o_. This shift in fluorescence intensity may be due to changes in fall in the optical properties of the different leaves induced by senescence, like changes in the pigment composition or the structure of the thylakoids.

The initial velocity of the fluorescence rise has not often been studied in plant stress experiments. The OJ phase reflects the speed of reduction of the primary acceptor Q, thus the more blocked QA is, the steeper is the initial fluorescence rise (Cicek et al. [5]. In early summer *Calamagrostis* showed a more rapid initial velocity, compared to *Alnus* and *Rhododendron*, with identical values for the two sites. In contrast *Rhododendron* showed a strong difference between the two sites, with better performance at lower nitrogen content. In fall the initial velocity in *Calamagrostis* at the two sites differed strongly, being 40% higher for the low nitrate site compared to close to the alder bushes. *Rhododendron* close to *Alnus* differs as well significantly from the other site. The performance of *Vaccinium* at the two sites is practically identical (p = 0.56). Again, the fall values for all species point to downgraded environmental conditions and plant senescence.

The level of the J-step is also a measure of stress, which is easily demonstrated in the kinetic data (Fig. 6). Nitrogen starvation also increased the J step [25], which has been discussed as a decrease in electron transport beyond the acceptor Q. In our alpine nitrogen gradient both *Calamagrostis* and *Rhododendron* react significantly on nitrate differences in the soil both in early August and mid-September. It must be noted that for *Calamagrostis* the J-value in summer is the only parameter which is statistically different for the two sites of different N-availability.

A positive K-band, located between the O and J step (Fig. 7), indicates a decrease of overall efficiency of activity of the oxygen evolving complex [9, 25, 28]. It is observed upon various stress situations. Under drought situations with barley the K-band changed from negative to positive, the stronger the stress acted upon the plants [21]. A positive K-band has also been observed in nitrogen fertilization experiments with crop plants after a lack of nutrients. *Calamagrostis* showed, both in early August (not shown) and mid-September, a strong positive difference between the two sites; interestingly the maximum was reached already after 300 μsec in September. In contrast, *Rhododendron* had a slightly positive signal in August which turned to a negative one in September. For *Vaccinium* the K-band was also negative. As nitrogen is a strong determinant of plant growth and up to 50% of leaf nitrogen is found in the photosynthetic structures, it is not astonishing that limited nitrogen supply for the densely growing *Calamagrostis* has severe effects on the sensitive oxygen-evolving system of the PSII with a high protein turnover. In the low nitrogen adapted shrubs *Rhododendron* and *Vaccinium* a strong K-band is missing.

The photosynthetic performance index PI has achieved an important measure of the overall vitality of the plant and contains information on the trapping of absorbed photons, on the activity of the reaction centers in photosystem II and the efficiency of the electron transport beyond PSII [14, 29, 31]. It is therefore highly sensitive to a multitude of environmental effects [13]. As a consequence, PI varies widely between different plant species, leaf age, season, the daily cycle and environmental effects, as well as for the same plant species between different sites [15, 23, 24, 36]. PI reacts also on various nutritional deficiencies including nitrogen [11]. As PI is positively correlated both with the nitrogen content of the leaves [20] and the rate of photosynthetic CO_2_-fixation [2], PI should be a good control for N-deficiency [11, 18, 25, 29]. It was therefore surprising, that PI for the 2 sites for *Calamagrostis* was statistically identical in early August, but changed to a strong difference mid-September with a higher value at higher nitrogen. Higher nitrogen in soil resulted in a higher PI also in *Rhododendron* in early August. In contrast, in September the situation was reversed. In *Vaccinium* PI at the 2 sites was statistically identical, thus *Vaccinium* seems to be rather insensitive to a small N-variation.

How do these alpine plants react upon varying nitrogen availability in soil? For *Calamagrostis*, the indicators extracted from the fluorescence data (except F_o_) mid-September indicated positive differences for higher nitrogen between the sites near *Alnus* and the shrub bushes, demonstrating that the grass reacts positively to higher levels of nitrogen. In contrast in early August, although differences were visible in the averaged fluorescence scans (Fig. 6a), all parameters except for the variable fluorescence F_J_ at the J-step, indicated no differences between low and high nitrogen. *Rhododendron* was also influenced by the nitrogen content in soil. Beginning in August, it performed better near *Alnus*, while in September the situation became reversed. All parameters point to less photosynthetic efficiency near the *Alnus* site. During the summer growth period N-availability was more important than mid September with already harsh climatic conditions above 2000 m a.s.l. *Vaccinium* seemed to be less sensitive towards changes in nitrogen; however, it must be noted that it was absent close to the *Alnus* front.

As many environmental factors affect parameters derived from fast fluorescence, one could question whether the described changes here are due specifically to the nitrate concentration in soil. However, as the distance between alder bushes and the dwarf shrub region is about 10 m, different climatic effects are not plausible. Furthermore, the soil samples from the different sites (b-1 to b-5) are of similar structure, inhomogeneous and rich in coarse particulate dead plant organic matter. It is assumed that in this rather small habitat, the plants are well adapted to the present local prevailing environmental conditions and stay over the season in a slowly reacting dynamic equilibrium, including the nitrogen flow from the alder symbionts.

When the results of the two sampling dates are compared it is obvious that plant vitality is significantly better in summer. For *Alnus* PI dropped in fall by 65%, a decline that holds also for F_v_/F_m_. The velocity of the initial rise and the position of the J-step increased. These indicators changed likewise in *Calamagrostis* and *Rhododendron* between early August and mid-September. Dropping temperatures and altered light conditions on the north-exposed hillside must have affected all plants, but certainly also had an impact on the nitrogen-fixation rate of the *Alnus* symbionts. This would then have altered the N-gradient and thus the N-supply for the grass and shrubs at the different sites. It must be noted that between the two sampling dates several strong drops in ambient temperature occurred as recorded in nearby meteorological stations (Swiss Meteo 2018).

## Conclusions

The change in plant diversity in a small area away from *Alnus* bushes and trees suggested a strong dependency on the nitrogen supply in soil provided by the nitrogen-fixing capacity of the root symbionts *Frankia alni*. This was confirmed by chemical nitrate analyses and strengthened by the distribution of the mean N-indicator values of plots in the transects showing a gradient away from the *Alnus*-covered area. Prompt fluorescence OJIP measurements indicated that at lower nitrogen levels in the soil, the same plant species often showed signs of nutrient stress, such as more rapid reduction of the primary acceptor, a reduced capacity on the donor site of photosystem II and a lower performance index. This natural ecosystem was under quasi equilibrium conditions and slow changes in the seasonal cycle were taking place during this study. Fast fluorescence data showed differences in the photosynthetic metabolism within the same plant species when sites with high and low nitrogen soil concentration were compared. Not all species were equally sensitive. While the grass *Calamagrostis* reacted strongly to changes in soil nitrogen, *Vaccinium* was rather insensitive.

## Materials

### Site description

The experimental site is located in the Val Piora (Ticino, Switzerland) (N 46° 32.671 E 8° 42.974, 2040 m a.s.l.); the geographical location can be verified under//map.geo.admin.ch, using the search name “Alpe di Piora (TI)-Quinto”. A roughly rectangular N-E oriented area of about 30 × 40 m in size with a slope of about 30°–35° is surrounded by *Alnus viridis* bushes and trees. A defined strip of grasses of 6 to 8 m width follows adjacent to the *Alnus* border (see Figs. 1, 2), consisting of mainly *Calamagrostis varia*. This strip then abruptly merges with dwarf shrubs dominated by *Rhododendron ferrugineum* and *Vaccinium uliginosum*. As soil structure and physical parameters are similar over the site, these distinct changes in plants composition may be based on the differences in nutrient content in the soil. A schematic drawing of the site is given in Fig. 2.

### Sampling, plant abundance

The substratum consisted of ~ 10–40 cm tall humus layer above Bündner shist (Penninicum). A horizontal and a vertical transect was selected within the area (Fig. 2) to characterize the species diversity. Each transect consisted of six plots or collection squares of 1 × 1 m at every 4 to 5 m. For every square the composition of species and their frequency were recorded on the 2nd of August 2017 following the cover abundance scale of Braun-Blanquet [1] (Table 1).

### Determining indicator values

Landolt [16], Landolt et al. [17] and Ellenberg et al. [8] characterized many plant species empirically by a specific nitrogen (N) indicator value. In this investigation we used the indicator values of Landolt et al. [17] which are based on a simple ordinal classification of plants according to the position of their realized ecological niche using the ordinal scale of 1 (= very low soil N) to 10 (= very rich soil N). Thimonier et al. [32] used these indicators for monitoring the ground vegetation in forests. These index values for the plant species found are listed in column 1 of Additional file 1: Table S1. For all plant species present these indicator values for nitrogen were combined with the Braun-Blanquet frequencies of each species in each 1 m^2^ plot resulting in a mean indicator value for each plot. These values are shown for each plot in Fig. 3. The detailed calculation is given for plot 1 in Additional file 1: Table S1.

### Nitrate determination in soil

Using a spatula, five samples of soil of about 100 g were collected on the 13th September 2017 from the top 20 cm of each zone in the horizontal transect and from a control site distant from *Alnus* trees. Sampling sites are indicated in Fig. 2 (b-1, b-2, b-3, b-4). Sample b-5 was collected in the dwarf shrub vegetation about 100 m distance from *Alnus* vegetation.

For the nitrate determination, 5 separate aliquots of 15 to 50 g of fresh soil from each sample (b-1 to b-5) were manually homogenized and suspended in 100 ml 0.01 M CaCl_2_, stirred for 60 min at RT (25 °C) on a lab shaker (Kühner) at 140 rpm, then filtrated through a Whatman 790 circular filter. The filtrate was directly used for the nitrate determination by a Skalar’ Continuous Flow-Analyzer by measuring the absorbance at 540 nm according to the method designed by the Skalar company (Breda, The Netherlands). Nitrate was the only N species detected in the extracts. After extraction the dry weight of the samples was determined, and the nitrate concentration was calculated per dry weight of soil, taking the Flow-Analyzer volumes of each extract into account.

### Chlorophyll-*a* fluorescence

Chlorophyll-*a* fluorescence was analyzed using the portable fluorometer Pocket PEA (Hansatech, King’s Lynn, England) on the 2nd of August 2017 and the 13th September 2017 in the early afternoon. From the different plant samples found at the site, the following dominant species were selected: *Alnus viridis, Rhododendron ferrugineum, Vaccinium uliginosum, Calamagrostis varia* and *Avenella flexuosa.* Light-exposed, fully developped leaves were randomly selected from an area of 50 × 50 cm (*Calamagrostis, Avenella*), respectively from several small shrubs (*Rhododendron, Vaccinium*) and from the outside of the larger bushes (*Alnus*). Plant material was sampled at the vegetation boundaries as determined for nitrate determination (b-1, b-2, b-3, b-4). Detached leaves were fixed in commercial leaf clips (Hansatech, England) and kept in the dark for 20 min prior to the fluorescence measurement [10, 31].

At each site (b-1 to b-4) fast fluorescence was measured with 5 leaves from different plants of the same species located at the same site. These experiments were repeated 3 times within 30 min, leading to 15 measurements per plant and site.

Excitation intensity was 3500 µmol photons m^−2^ s^−1^ with red light of 650 nm for 3 s. From the fluorescence induction signal with high temporal resolution from 10 µs to 3 s, the instrument determined initial (F_o_) and maximum (F_m_) fluorescence and calculated the variable fluorescence (F_v_) at specified time intervals, and also provided further specific parameters such as the potential quantum yield of PS II (F_v_/F_m_), or the performance index (PI_ABS_). For further calculations and the visualization, the experimental data were transferred into Excel sheets to calculate the mean value of all data points of each time course from 15 replicates of the same plant species and location. To reduce the variability due to e.g. leaf age and leaf position and local soil differences, the fluorescence induction curves were normalized by setting F_o_ = 0 and F_m_ = 1. The result is illustrated in Fig. 5. The initial velocity was calculated as (F_0.15ms_–F_0_)/0.15.

### Statistical treatment

To calculate mean values, standard deviation and significance with t-test analysis the software MaxStat was used. All data points presented in the figures are means of 5 consecutive measurements which were repeated 3 times (n = 15) in intervals of 30 min. Additional file 2: Table S2 reports the statistical data of the nitrate concentrations.

## Supplementary information

**Additional file 1: Table S1.** Calculation of the mean N-value of each plot from the indicator values of Landolt et al. [17] and its frequency based on Braun-Blanquet [1]. For each plant (in this example of plot 1 (Table 1)) the Braun-Blanquet frequency was increased by 1 to obtain values from 1 to 5. This number was multiplied by the Landolt-indicator value. The mean N-value per plot results in the sum of these products divided by the sum of the new frequency values. The calculation steps are given below.

**Additional file 2: Table S2.** Statistical proof of significance (p-values after Mann–Whitney, MaxStat-Lite, 3.60), 5 measurements per site b-1 to b-5.

## Data Availability

The PEA data files can be obtained from the corresponding author.

## Abbreviations

OJIP: Fast fluorescence rise from F_o_ to F_m_
F_o_: Initial fluorescence O
F_m_: Maximal fluorescence P after 1 s
F_v_: Variable fluorescence, = (F_m_-F_o_)
PI: Photosynthetic performance index
J-step: Variable fluorescence at 2 ms, = F_J_
RC: Reaction center
PS II: Photosystem II
PS I: Photosystem I
K-band: Selected part of OJIP between O and J steps
PEA fluorimeter: Photosynthetic efficiency analyzer
