# Supplementary material for: Alpine ecology, plant biodiversity and photosynthetic performance of marker plants in a nitrogen gradient induced by Alnus bushes
Source: BMC Ecol. 2020 Apr 20;20:23. doi: 10.1186/s12898-020-00292-9 (PMC7171859; doi:10.1186/s12898-020-00292-9)
Supplement: Supplementary file 1 — Additional file 1: Table S1. Calculation of the mean N-value of each plot from the indicator values of Landolt et al. [17] and its frequency based on Braun-Blanquet [1]. For each plant (in this example of plot 1 (Table 1)) the Braun-Blanquet frequency was increased by 1 to obtain values from 1 to 5. This number was multiplied by the Landolt-indicator value. The mean N-value per plot results in the sum of these products divided by the sum of the new frequency values. The calculation steps are given below. [file 12898_2020_292_MOESM1_ESM.doc]

**Additional file**

Table S1:

Calculation of the mean N-value of each plot from the indicator values of Landolt et al. (2010) and its frequency based on Braun-Blanquet (1964). For each plant (in this example of plot 1 (Table 1)) the Braun-Blanquet frequency was increased by 1 to obtain values from 1 to 5. This number was multiplied by the Landolt-indicator value. The mean N-value per plot results in the sum of these products divided by the sum of the new frequency values. The calculation steps are given below.

|  | N-Indicator value of Landolt | Plot 1, Frequency (Braun-Blanquet) | New N-  value | Calculation | Number of counts |
| --- | --- | --- | --- | --- | --- |
| Species |  |  |  |  |  |
| *Alnus viridis* | 4 | 4 | 4+1 | 5*4=20 | 5 |
| *Rhododendron ferrugineum* | 2 | + | 1 | 1*2= 2 | 1 |
| *Vaccinium myrtillus* | 2 | 1 | 1+1 | 2*2=4 | 2 |
| *Veratrum album* | 4 | 1 | 1+1 | 2*4=8 | 2 |
| *Dryopteris dilatata* | 3 | + | 1 | 1*3=3 | 1 |
| *Oxalis acetosella* | 2 | + | 1 | 1*2=2 | 1 |
| *Calamagrostis varia* | 2 | 1 | 1+1 | 2*2 =4 | 2 |
| Sum |  |  |  | 43 | 14 |
| **Mean N-Value** |  |  |  | 43/14 | 3.02 |
