# Supplementary material for: Alpine ecology, plant biodiversity and photosynthetic performance of marker plants in a nitrogen gradient induced by Alnus bushes
Source: BMC Ecol. 2020 Apr 20;20:23. doi: 10.1186/s12898-020-00292-9 (PMC7171859; doi:10.1186/s12898-020-00292-9)
Supplement: Supplementary file 2 — Additional file 2: Table S2. Statistical proof of significance (p-values after Mann–Whitney, MaxStat-Lite, 3.60), 5 measurements per site b-1 to b-5. [file 12898_2020_292_MOESM2_ESM.doc]

Table S2

Statistical proof of significance (p-values after Mann-Whitney, MaxStat-Lite, 3.60), 5 measurements per site b-1 to b-5.

| Sampling site | b-1 | b-2 | b-3 | b-4 | b-5 |
| --- | --- | --- | --- | --- | --- |
| b-1 | **1** | **0.46** | **0.12** | **0.01** | **0.01** |
| b-2 |  | **1** | **0.03** | **0.01** | **0.01** |
| b-3 |  |  | **1** | **0.12** | **0.01** |
| b-4 |  |  |  | **1** | **0.12** |
| b-5 |  |  |  |  | **1** |
